# Applications of Haptic Technology, Virtual Reality, and Artificial Intelligence in Medical Training During the COVID-19 Pandemic

**DOI:** 10.3389/frobt.2021.612949

**Published:** 2021-08-12

**Authors:** Mohammad Motaharifar, Alireza Norouzzadeh, Parisa Abdi, Arash Iranfar, Faraz Lotfi, Behzad Moshiri, Alireza Lashay, Seyed Farzad Mohammadi, Hamid D. Taghirad

**Affiliations:** ^1^ Advanced Robotics and Automated Systems (ARAS), Industrial Control Center of Excellence, Faculty of Electrical Engineering, K. N. Toosi University of Technology, Tehran, Iran; ^2^ Department of Electrical Engineering, University of Isfahan, Isfahan, Iran; ^3^ Translational Ophthalmology Research Center, Farabi Eye Hospital, Tehran University of Medical Sciences, Tehran, Iran; ^4^ School of Electrical and Computer Engineering, University College of Engineering, University of Tehran, Tehran, Iran; ^5^ Department of Electrical and Computer Engineering, University of Waterloo, Waterloo, ON, Canada

**Keywords:** COVID-19 pandemic, medical training, haptic, virtual reality, artificial intelligence

## Abstract

This paper examines how haptic technology, virtual reality, and artificial intelligence help to reduce the physical contact in medical training during the COVID-19 Pandemic. Notably, any mistake made by the trainees during the education process might lead to undesired complications for the patient. Therefore, training of the medical skills to the trainees have always been a challenging issue for the expert surgeons, and this is even more challenging in pandemics. The current method of surgery training needs the novice surgeons to attend some courses, watch some procedure, and conduct their initial operations under the direct supervision of an expert surgeon. Owing to the requirement of physical contact in this method of medical training, the involved people including the novice and expert surgeons confront a potential risk of infection to the virus. This survey paper reviews recent technological breakthroughs along with new areas in which assistive technologies might provide a viable solution to reduce the physical contact in the medical institutes during the COVID-19 pandemic and similar crises.

## 1 Introduction

After the outbreak of COVID-19 virus in Wuhan, China at the end of 2019, this virus and its mutations has rapidly spread out in the world. In view of the fact that no proven treatment has been so far introduced for the COVID-19 patients, the prevention policies such as staying home, social distancing, avoiding physical contact, remote working, and travel restrictions has strongly been recommended by the governments. As a consequence of this global problem, universities have initiated policies regarding how to keep up teaching and learning without threatening their faculty members and students to the virus. Thus, the majority of traditional in-class courses have been substituted to the online courses. Notwithstanding the fact that the emergency shift of the classes have reduced the quality of education during the COVID-19 pandemics Hodges et al. (2020), some investigators have proposed ways for rapid adaption of the university faculty and the students to the situation and improve the quality of education Zhang et al. (2020).

Nevertheless, the case of remote learning is different in the medical universities as the learning process in the medical universities is not just rely on the in-class courses. As an illustration, the medical training in the traditional way is accomplished by a medical student through attending some training courses, watching how the procedure is performed by a trainer, performing the procedure under supervision of a trainer, and at the final stage, independently performing the procedure. In fact, the traditional method of surgery training relies on excessive presence of students in the hospital environments and the skill labs to practice the tasks on the real environments such as physical phantoms, cadavers, and patients and that is why medical students are called “residents”. Thus, the aforementioned traditional surgery training methodology requires a substantial extent of physical contact between medical students, expert surgeons, nurses, and patients, and as a result, the risk of infection is high among those people. On the other hand, the assistive technologies based on virtual reality and haptic feedback have introduced alternative surgical training tools to increase the safety and efficiency of the surgical training procedures. Nowadays, the necessity of reducing the physical contact in the hospital environments seems to make another motivation for those assistive technologies. Therefore, it is beneficial to review those technologies from COVID-19 motivation aspect.

In this paper, the existing assistive technologies for medical training are reviewed in a COVID-19 situation. While there are several motivations for those technologies such as increasing the safety, speed, and efficiency of training, the new motivations created for those technologies during the COVID-19 pandemic are the specific focus of this paper. In spite of the existing literature on COVID-19, our main focus is surgery training technologies that help to reduce physical contact during this and other similar pandemics. Notably, a number of those studies have analyzed systemic and structural challenges applicable to medical training programs with little emphasis on technological aspects of the subject Sharma and Bhaskar (2020), Khanna et al. (2020). On the other hand, the methods of remote diagnostics and remote treatment have received a great deal of attention after COVID-19 pandemic and a massive body of literature have covered those topics Tavakoli et al. (2020), Feizi et al. (2021), Akbari et al. (2021). In contrast, less studies have given special attention on remote training and remote skill assessment which is the subject of this paper. For this reason, this paper addresses scientific methods, technologies and solutions to reduce the amount of physical contact in the medical environments that is due to training reasons.

Relevant literature was chosen from articles published by IEEE, Frontiers, Elsevier, SAGE, and Wiley with special attention to the well-known interdisciplinary journals. The search was preformed using the keywords “remote medical training,” “skill assessment in surgery,” “virtual and augmented reality for medical training,” “medical training haptic systems,” and “artificial intelligence and machine learning for medical training” until June 30, 2021. The literature was examined to systematically address key novel concepts in remote training with sufficient attention to the future direction of the subject. Finally, it is tried to review the problem in the COVID-19 context in a way that the discussed materials are distinct from similar literature in a conventional non-COVID context.

The rest of this paper is organized as follows: The clinical motivations of the training tools are discussed in Section 2. The virtual and augmented reality and the related areas of utilization for medical training are described in Section 3. Section 4 explains how haptic technology may be used for medical training, while Section 5 describes some data-based approaches that may be used for skill assessment. Then, the machine vision and its relevant methods used for medical training are presented in Section 6. Finally, concluding remarks are stated in Section 7.

## 2 The Clinical Motivation

The process of skill development among medical students have always been a challenging issue for the medical universities, as the lack of expertise may lead to undesired complications for the patients Kotsis and Chung (2013). Moreover, owing to the rapid progress of minimal invasive surgeries during the past decades, the closed procedures have been becoming a method of choice over traditional open surgeries. In the minimal invasive surgery, the instruments enter the body through one or more small incisions, while this type of surgery is applicable to a variety of procedures. The foremost advantage of this technique is the minimal affection to healthy organs, which leads to less pain, fewer post-operative complications, faster recovery time, and better long-term results.

However, the closed surgery technique is more challenging from the surgeon’s point of view since the surgeon does not have a complete and direct access on the surgical site and the tiny incisions limit the surgeon’s accessibility. Owing to the limited access, some degrees of freedom are missing and surgeon’s manipulation capability is considerably reduced. Furthermore, there is fulcrum effect at the entry point of the instrument, i.e., the motion of the tip of the instrument, which is placed inside the organ, and the external part of the instrument, which is handled by the surgeon, are reversed. This results in more difficult and even awkward instrument handling and requires specific and extensive surgical training of the surgeon. As a result, the minimal invasive surgeries demands advanced expertise level, the lack of which might cause disastrous complications for the patient. These conditions are equally important in many medical interventions, especially in minimally invasive surgeries. Here a number of specific areas of surgical operation are expressed in order to address complications that might occur during the training procedures.

• Eye surgery:

An important category of medical interventions which need a very high skill level is intraocular eye surgical procedures. Notably, the human eye is a delicate and highly complex organ and the required accuracy for the majority of intraocular surgeries is in the scale of 50–100 microns. The closed type of surgery is applicable to a number of eye surgeries such as the Cataract surgery in the anterior segment as well as the vitro-retinal surgical procedures in the posterior segment. Notably, some complications such as Posterior Capsule Rupture (PCR) for cataract surgery and retina puncture for the vitro-retinal surgical procedures are among the relatively frequent complications that might happen, due to the surgeon’s lack of surgical skills and dexterity. It is shown in a study on ophthalmic residents that the rate of complications such as retinal injuries is higher for the residents with less skills Jonas et al. (2003).

• Laparoscopic Cholecystectomy

Another example is Laparoscopic Cholecystectomy (LC) which is now the accepted standard procedure across the world and is one of the most common general and specialist surgical procedures. However, it can be prone to an important complication that is bile duct injury (BDI). Although BDI is uncommon but it is one of the most serious iatrogenic surgical complications. In extreme BDI cases, a liver resection or even liver transplantation becomes necessary. BDI is considered as an expensive medical treatment and its mortality rate is as high as 21% Iwashita et al. (2017).

• Neurosurgery

Neurosurgery is another field that deals with complex cases and requires high accuracy and ability in the surgeon’s performance. In a prospective study of 1,108 neurosurgical cases, 78.5% of errors during neurosurgery were considered preventable Stone and Bernstein (2007). The most frequent errors reported were technical in nature. The increased use of endoscopy in neurosurgery introduces challenges and increases the potential for errors because of issues such as indirect view, elaborate surgical tools, and a confined workspace.

• Orthopedic surgery

In the field of orthopedics, knee and shoulder arthroscopic surgeries are among the most commonly performed procedures worldwide. There is a steep learning curve associated with arthroscopic surgery for orthopaedic surgery trainees. Extensive hands-on training is typically required to develop surgical competency. The current minimum number of cases may not be sufficient to develop competency in arthroscopic surgery. It is estimated that it takes about 170 procedures before a surgeon develops consultant-level motor skills in knee arthroscopic surgery Yari et al. (2018). With work-hour restrictions, patient safety concerns, and fellows often taking priority over residents in performing cases, it is challenging for residents to obtain high-level arthroscopic skills by the end of their residency training.

**FIGURE 1:**
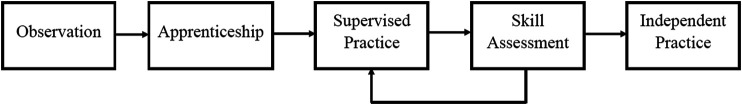
Process of procedural skill development in medical training and surgery.

The above motivation shows the importance of skill development among the medical students. The standard process of procedural skill development in medicine and surgery is shown as a diagram in Figure 1. In the observation stage, the medical students need to attend a clinical environment and watch how the procedure is performed by a trainee. Then, the medical students get involved in the operation as an apprentice, while the actual procedure is performed by the trainer. Later, the medical students practice the operation under direct supervision of the trainer, while the trainer assesses the skill level of the medical students. The supervised practice and skill assessment steps are repeated as long as the trainee does not have enough experience and skill to conduct the procedures without supervision of the trainer. Finally, after obtaining sufficient skill level, the trainee is able to independently perform the operation.

Remarkably, a learning curve is considered for each procedure, which means that performance tends to improve with experience. This concept applies for all of the medical procedures and specialties, but complex procedures, surgery in particular, are more likely to gradual learning curves, which means that improvement and expertise is achieved after longer training time. Some of the important factors in the learning curve are manual dexterity of the surgeon, the knowledge of surgical anatomy, structured training and mentoring and the nature of the procedure. The learning curve is longer for minimally invasive procedures than that for open surgical procedures. The learning curve is also influenced by the experience of the supporting surgical team. Besides, learning curves depend on the frequency of procedures performed in a specified period. Many studies suggest that complication rates are inversely proportional to the volume of surgical workload.

Notably, the above mentioned process of skill development require a considerable extent of physical contact between the trainees, the expert surgeons, the nurses, and the patients, while this shall be reduced in the COVID-19 pandemic. In addition to the high risk of infection in the medical universities with the conventional medical training approaches, the majority of the health-care capacity is focused on fighting the COVID-19 virus and consequently, the education requirements of medical universities are failed to be entirely fulfilled. As a result, the training efficiency of medical universities will be reduced, provided that they just rely on the conventional training approaches. This will have possible side-effects on the future performance of the health-care system mainly due to the insufficient number of recently graduated students with adequate expertise level.

On the other hand, traditional education takes place in hospitals and on real patients, which face several problems during the COVID-19 pandemic: the hospital environment is contaminated with the virus, hospital staff and physicians are very busy and tired and have less training capacity, prolonged hospital stays of patients to train students put them at greater risk for exposure to the virus, especially if complication occurs by a resident who does not have gained sufficient skills during the training procedure. Therefore, training with assistive devices outside the hospital may play an effective role in this situations. The highlighted factors can significantly be improved by assisted learning, especially in minimally invasive procedures. In more complex surgeries, the complications becomes more serious, the learning curve will be longer, and the role of assisted learning becomes more prominent.

To solve the above mentioned problems, assistive training tools provide a variety of solutions through which the medical universities are able to continue their education procedures, while the risks enforced by the COVID-19 outbreak are reduced. In the following sections, the main assistive training tools including the haptic systems, virtual reality, machine vision, and data mining are reviewed and the areas in which those technologies facilitate the training process during the COVID-19 pandemic are detailed. The aim of these technologies is to have the training efficiency higher or at least equal to that of the conventional training methods without risk of infection of the involved parties to the virus.

## 3 Virtual and Augmented Reality

Virtual Reality is employed to create an immersive experience for various applications such as visualization, learning and education. In virtual reality, a computer generated graphical presence is visualized using a head mounted display and the user can interact with 3D objects located in the virtual world. In addition to VR, the Augmented Reality (AR) is developed to add 3D objects to the real world creating a different experience by adding digital information to the real objects in the surrounding environment. Although experiencing the 3D objects in VR scenes is far from the interaction with real objects, the VR experience is getting closer to the real world environments by the help of more realistic computer graphics and full-body haptics suits.

The virtual reality (VR) and augmented reality (AR) are getting more interest as a training technique in the medical fields, unlocking significant benefits such as safety, repeatability and efficiency Desselle et al. (2020). Furthermore, during the COVID-19 pandemic, remote training and consulting are considered as vital advantages of VR/AR based training methods (Singh et al., 2020).

**FIGURE 2:**
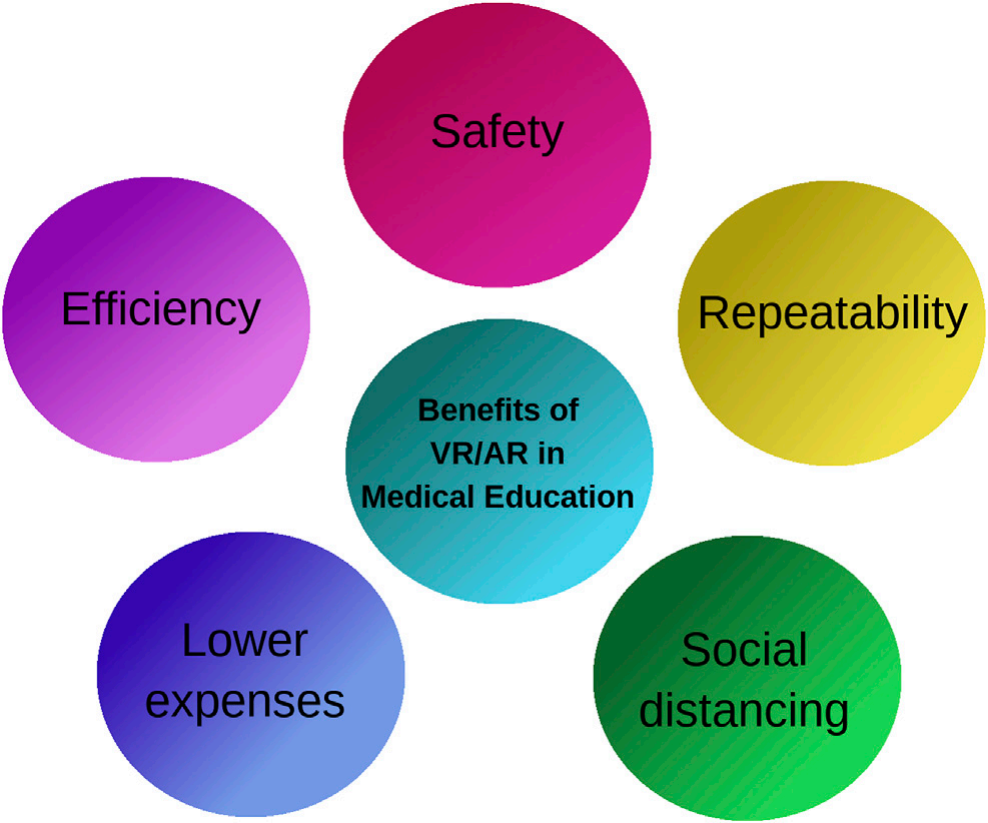
VR/AR advantages in medical training.

Some advantages of using VR/AR in medical training are depicted in Figure 2. Safety is the first and the most important benefit of VR/AR employment in medical education. Complex medical operations may be performed in a simulated environment based on VR with complete safety and without putting the patient’s life into danger. Repeatability is the second advantage of using VR as any simulation scenario in the field of medical training can be repeated over and over until the trainee is completely satisfied. During the COVID-19 pandemic it is vital to practice social distancing which is delivered by VR/AR employment in medical education. Medical training and surgery simulation by computer graphics in VR/AR virtual environments results in reduced training costs as no material except than a computer, a VR headset and a haptic device is required. Since medical training by VR/AR is performed using a computer, the surgery simulation is always in hand as soon the computer and VR headset are ready to be used. Therefore, the efficiency of medical training is increased as no time is required for either preparation of an operation room or getting a patient ready.

**FIGURE 3:**
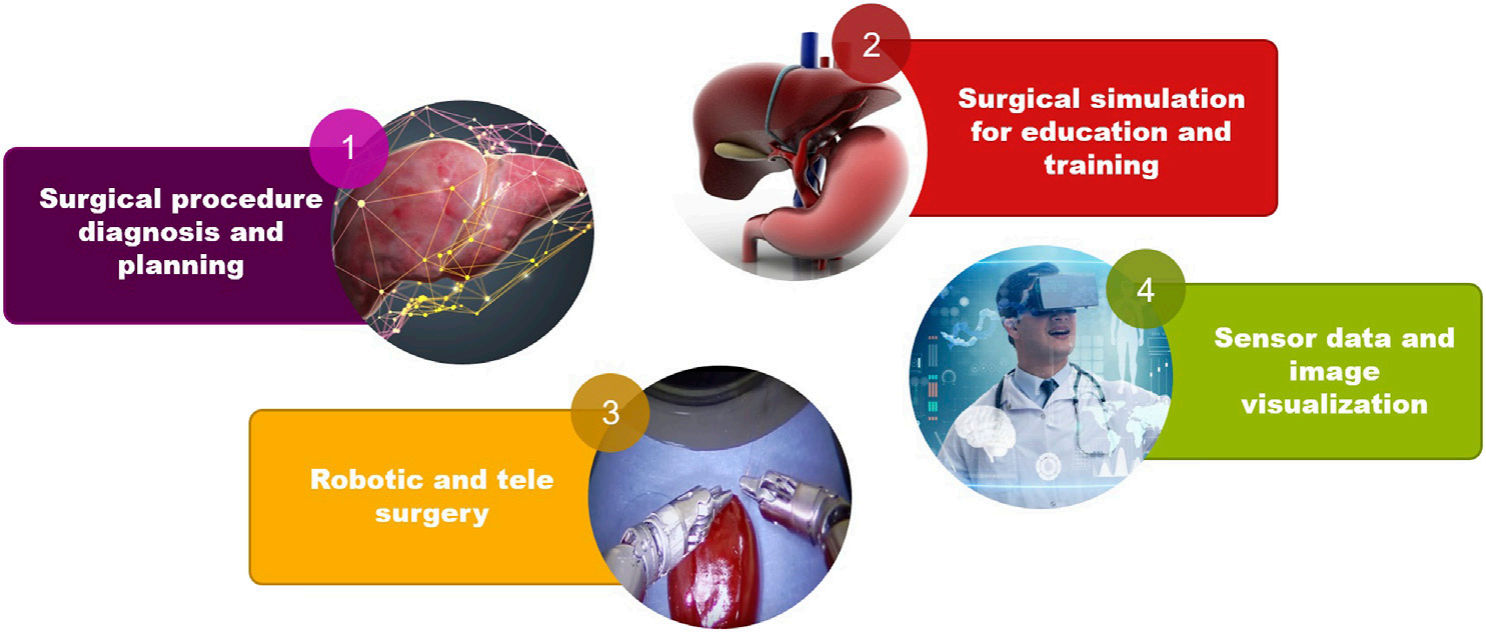
VR/AR applications in surgical training.

VR/AR techniques are employed in various applications in surgical training as it can be seen in Figure 3. The first application of AR/VR in surgical training is surgical procedure diagnosis and planning. Using AR/VR, the real surgical operation is simulated ahead without putting the patient’s life into danger. The AR/VR is used in surgical education and training which is mentioned as the second application. Simulation based environments are developed for training of medical students by virtual human anatomy 3D models. Another application of AR/VR is robotic and tele-surgery, by which surgical consulting becomes possible even from a far distance. The last application of AR/VR in surgical training is sensor data and image visualization during the surgical operation which makes the effective usage of patient’s medical data possible.

It is shown that the learning curve of hip arthroscopy trainees is significantly improved using a virtual reality simulator (Bartlett et al., 2020). In this study, a group of twenty five inexperienced students were chosen to perform seven arthroscopies of a healthy virtual hip joint weekly. The experimental results indicated that average total time decreased by nearly 75*%* while the number of collisions between arthroscope and soft-tissues decreased almost by 90*%*.

VR is also employed in orthopedic surgical training, where 37 residents participated in a study to obtain an understanding of the LISS^1^ plating surgical process (Cecil et al., 2018). The developed virtual surgical environment is equipped with a haptic device to perform various activities such as assembling LISS plate, placing the assembled LISS plate correctly inside the patient’s leg, and attaching the LISS plate to the fractured bone. The test was divided into pre–test where the students get familiar with the surgery process and the post–test which is devoted to the actual evaluation phase. The participants had 1 h to finish both the pre–and post–tests which resulted in improvement of learning the LISS plating surgical process.

The applicability and effectiveness of VR based training in orthopedic education is evaluated in (Lohre et al., 2020), where nineteen orthopedic surgical residents cooperated in this study. The surgical residents performed a glenoid exposure module on a VR based simulator using a haptic device as the input controller. The result of training of residents using VR simulator has been compared to the conventional surgery training methods. Considering the learning time, repeating 3 to 5 VR based surgery experiments by the residents, resulted in 570*%* training time reduction. Additionally, VR based surgical training helped the residents to finish glenoid exposure significantly faster than the residents trained by conventional education methods.

Orthognathic surgery is another surgery field considered for VR based training as it is one of the complex surgical procedures (Medellin-Castillo et al., 2020). While conventional OSG^2^ learning techniques are dependent to cadavers or models and experienced surgeons are trained after several years of experiments in operating rooms, employment of VR in surgical training can reduce the learning time and the education cost at the same time. In this study, three cases are considered for evaluation of VR in OSG, cephalometry training, osteotomy training and surgery planning to be precise. The experimental results indicated that the combination of haptics and VR is effective in skill improvement of trainees and surgery time reduction. Furthermore, the surgery errors and mistakes are reduced by using haptic feedback to recreate the sense of touch as trainees can detect landmarks more precisely in comparison to conventional techniques.

In conjunction with VR, the AR technology has also been used in various medical fields for training such as neurosurgical training (Si et al., 2019). Anatomical information and other sensory information can be visualized to the surgeons more properly, and therefore, more accurate decision can be made during a surgery. Although this study is only applicable to the simulated environments because of registration problem, the experiment indicated the effectiveness of the simulator in skill improvement of surgeons.

While key features of VR/AR have led to improved training specially in surgical training, there are some limitations that should be considered (kumar Renganayagalu et al., 2021). The first limitation of VR simulators is the cost of VR content production, and therefore, most of simulators are made for very specific type of simulation in a limited context. The second limitation is the immaturity of interaction devices for VR simulations, which has a great affect on the user experience. Another limitation of VR usage in medical training is the inability of using VR devices for long period of time as the VR devices are made for entertainment and not for a long training session.

It can be concluded that in spite of some limitations, VR/AR based simulators equipped with a haptic device can be used in medical surgery training in order to achieve skill improvement and training time reduction. Furthermore, during the isolation requirements due to COVID-19 pandemic, VR/AR based techniques can be well employed for medical training.

## 4 Teleoperated Haptic Systems

**FIGURE 4:**
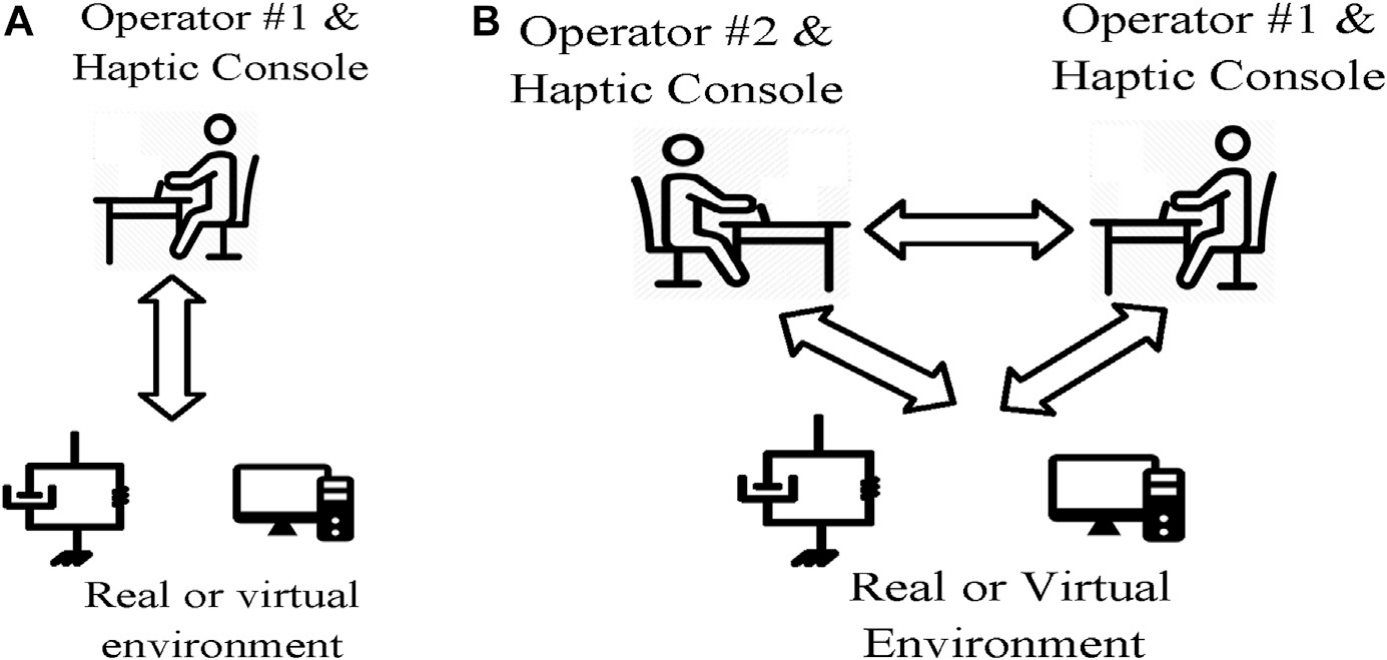
Single user vs. dual user haptic systems. **(A)** Single user haptic system. **(B)** Dual user haptic system.

Haptic systems provide the sense of touch with remote objects without the need of actual contact. It also provides collaboration between several operators without the need of any physical contact. As depicted in Figure 4, based on the number of the operators, the haptic systems may be classified into single user, dual-user or multi-user haptic systems. Single user haptic systems enable a single human operator to interact with a remote or virtual environment, whereas dual-user or multi-user haptic systems provide a mechanism for collaboration of two or multiple human operators. The medical training applications of those systems is presented here.

### 4.1 Single User Haptic Systems

Single user haptic systems extend the abilities of human operators to interact with remote, virtual, and out-of-reach environment. In the field of surgery training, a number of investigations have proposed haptic training simulators for training of minimally invasive surgery (MIS) Basdogan et al. (2004), dental procedures Wang et al. (2014), sonography Tahmasebi et al. (2008), and ocular therapies Spera et al. (2020). As shown in Figure 4A, a typical single-user haptic simulator system consists of a human operator, a haptic interface, a graphical interface, and a reference model for the virtual object. Notably both the graphical interface and the haptic interface utilize the reference model to provide necessary feedback for the operator. While the graphical interface provides a visual feedback of the environment, the haptic interface provides the kinesthetic feedback of the interaction between the tool and the surgical field. Indeed, the role of haptic feedback is to recreate the sense of contact with the virtual environment for the operator. As a result, the circumstances of actual operation is provided for the medical students, while the need of physical presence in the clinical environments is eliminated. Indeed, through haptic technology, the medical students are able to practice on a virtual environment without the need of presence at the clinical environment. Thus, the risk of infection during the COVID-19 pandemic is effectively reduced.

### 4.2 Dual User Haptic Systems

The cooperative and joint conduction of an operation either for the purpose of collaboration or training, as a fundamental clinical task, cannot be provided by single user haptic systems. In order to make the cooperation of two surgeons possible, the system should be upgraded to a dual user haptic system by adding another haptic console. A dual user haptic system is a more recent advancement in haptic technology, and it consists of two haptic consoles, one for the trainer and one for the trainee Shahbazi et al. (2018a). Remarkably, the traditional collaboration methods require direct physical contact of the persons conducting the operation, whereas the haptic-based collaboration approach eliminates the physical contact of the collaborators. As a result of removing the need of physical contact, the involved people are no longer in the risk of the Corona virus. A commercial dual user haptic system developed by intuitive Surgical Inc. ^®^ is the da Vinci Si Surgical System which supports training and collaboration during minimally invasive surgery. The da Vinci Si System builds on the existing da Vinci technology, where it has a number of enabling features such as leading-edge 3D visualization, advanced motion technology, and sufficient dexterity and workspace. However, the da Vinci Si does not provide active supervision and intervention of the trainer on the trainee’s actions. As an illustration, in the case that the trainee controls the procedure, the trainer does not have the possibility to guide the trainee during the procedure.

The issue of supervision and intervention of the trainer during the operation in dual user haptic systems have been a topic of active investigation during the past years. A number of studies have utilized the concept of *dominance factor* to determine the task dominance of each operator Nudehi et al. (2005), Khademian and Hashtrudi-Zaad (2012), Shahbazi et al. (2014b), Motaharifar et al. (2016). In those approaches, the trainee is given a partial or full task authority by the trainer based on his/her level of expertise. Notably, the task authority provided by these control architectures is supposed to be fixed during the operation. Thus, changing the authority of the surgeons and specially blocking the trainee’s commands is not possible in the middle of the operation. This might lead to undesired operative complications specially in the case that the trainee makes a sudden unpredictable mistake.

Fortunately, a number of investigations have developed control architectures to address the above shortcoming of the previously proposed haptic architectures Motaharifar et al. (2019b), Shahbazi et al. (2014a), Motaharifar and Taghirad (2020). As a case in point, an S-shaped function is proposed in Motaharifar et al. (2019b) for the adjustment of the corrective feedback in order to shape the trainee’s muscle memory. In fact, the training approach behind the presented architecture is based on allowing the trainee to freely experience the task and be corrected as needed. Nevertheless, through the above scheme, the trainee is just granted the permission to receive the trainer’s motion profile; that is, the trainee is deprived of any realistic contribution to the surgical procedure. In contrast, several investigations have proposed mechanisms for adjusting the task dominance, through which the trainee is granted partial or full contribution to the task Shahbazi et al. (2014a), Motaharifar and Taghirad (2020), Liu et al. (2015), Lu et al. (2017), Liu et al. (2020). Remarkably, the above approaches require both the trainer and the trainee to completely perform the operation on their haptic devices, and the actual task authority is determined based on the position error between the trainer and the trainee Shahbazi et al. (2014a), Motaharifar and Taghirad (2020), Liu et al. (2015), Lu et al. (2017), Liu et al. (2020). This constitutes an important limitation of the above architectures, since the trainer is enforced to be involved in every detail of each operation and even the trivial ones. Notably, the trainer’s obligation to precisely perform every part of the surgical procedure has little compatibility with the trainer’s responsibilities in terms of supervisory assistance and interference. In fact, by grabbing the idea from the conventional training programs of the medical universities, the haptic architecture should be developed in such a manner that the trainer is able to intervene only in order to prevent a complication to the patient due to the trainee’s mistake. The issue of trainer’s supervisory assistance and interference is addressed in Motaharifar et al. (2019a) by adjusting the task authority based on the trainer’s hand force Motaharifar et al. (2019a). That is, the trainer is able to grant the task authority to the trainer by holding the haptic device loosely or overrule the trainee’s action by grasping the haptic device tightly. Therefore, the active supervision and interference of the trainer is possible without the need of any physical contact between the trainer and the trainee.

Although the above investigations address the essential theoretical aspects regarding dual user haptic systems, the commercialization of collaborative haptic system needs more attention. In the past years, some research groups have developed pilot setups of dual user haptic system with the primal clinical evaluation that have the potential of commercialization. For instance, the ARASH-ASiST system provides training and collaboration of two surgeons and it is preliminary designed for Vitreoretinal eye surgical procedures ARASH-ASiST (2019). It is expected that the commercialization and widespread utilization of those assistive surgery training tools is considerably beneficial to the health-care systems in order to decrease the physical contact during the COVID-19 pandemic, and to increase the safety and efficiency of training programs during and after this crisis.

Notwithstanding the fact that teleoperated haptic systems provide key benefits for remote training during COVID-19 pandemic, they face a number of challenges that inspire perspectives of future investigations. First, the haptic modality is not sufficient to recreate the full sense of actual presence at the surgical room near an expert surgeon. To overcome this challenge and increase the operators telepresence, the haptic, visual, and auditory components are augmented to achieve a multi–modal telepresence and teleaction architecture in Buss et al. (2010). The choice of control structure and clinical investigation of the above multi–modal architecture is still an area of active research Shahbazi et al. (2018b), Caccianiga et al. (2021). On the other hand, the on-line communication system creates another challenge for the haptic training systems. Notably, owing to the high-bandwith requirement for an appropriate on-line haptic system, the majority of existing haptic architectures in applications such as collaborative teleopertion, handwriting and rehabilitation cover off-line communication Babushkin et al. (2021). However, due to the complexity, uncertainty, and diversity of the surgical procedures, the online feedback from the expert surgeon is necessary for a safe and efficient training. The advent of 5G technology with faster and more robust communication network may provide enough bandwidth for an effective real-time remote surgery training.

## 5 Data Driven Scoring

A vital element of a training program is how to evaluate the effectiveness of exercises by introducing a grading system based on participants’ performance. The conventional qualitative skill assessment methods require physical contact between the trainer and the trainee since they are based on direct supervision of the trainer. On the other hand, the systematic approaches for skill assessment are based on collecting the required data using appropriate instruments and analyzing the obtained data, while they eliminate the requirement of physical contact between the trainer and the trainee. Thus, reviewing the systematic data-based methods is of utmost importance, as they can be utilized to reduce the physical contact during the COVID-19 Pandemic. In this section, some of the state of the art methods in surgical skill evaluation are reviewed. Following the trend of similar research in the context of surgical skill evaluation, we categorize the reviewed methods by two criteria. The first is the type of data, and the method uses for grading the participant. The second criterion is the features extraction techniques that are used during the evaluation stage.

Generally speaking, two types of data may be available in Robotic-Assisted surgery; kinematic and video data. Kinematic data is available when a robot or haptic device is involved. The most common form of capturing kinematic information is using IMUs, encoders, force sensors, magnetic field positioning sensors, etc. The video is generally recorded in all minimally invasive surgeries using endoscopy procedures.

Kinematic data are more comfortable to analyze because the dimensionality of kinematic data is lower than video data. Moreover, Kinematic information is superior to video in measuring the actual 3D trajectories, and 3D velocities Zappella et al. (2013). On the other hand, video data is more convenient to capture since no additional equipment and sophisticated sensors are needed to be attached to the surgical tool. Additionally, video data reflects the contextual semantic information such as the presence or absence of another surgical instrument, which can not be derived from the kinematic data Zappella et al. (2013). To use the video data effectively, one should overcome some common obstacles like occlusion and clutter. Using multiple cameras, if possible, can greatly assist in this procedure Abdelaal et al. (2020). In conclusion, it can be said that each type of data has its own merits and limitations, and using kinematic data as well as the video may result in a richer dataset.

Other than the kinematic and video data, another source of information is often disregarded in the literature. The expert surgeon who conducts the training program can evaluate the trainee’s performance and provide useful feedback regarding his/her performance. This type of information, which is at another semantic level compared to the sensory data, is called soft data. The hard and soft information fusion methods can merge the expert’s opinion with the kinematic and video data (hard data) to accomplish a better grading system.

Most surgical skill evaluation methods utilize a feature extraction technique to classify the participant’s skill level after acquiring the data, like expert, intermediate, and novice. The classification problem can be solved by employing some hand-engineered features or features that are automatically extracted from the data. Hand-engineered features are interpretable and easy to obtain. However, hand-engineered features are hard to define. Specifically, defining a feature that represents the skill level regardless of the task is not trivial. Therefore, the states of the art methods are commonly based on automatic feature extraction techniques. An end-to-end deep neural network is used to unfold the input data’s spatial and temporal features and classify the participant in one of the mentioned skill levels in an automated feature extraction procedure. While, Table 1 summarizes the topic of different data types and feature extraction techniques, we are going to cover some of the reviewed methods in the next sections.

**TABLE 1:** Summery of different sources of data and different feature extraction techniques.

	Data type		
	Kinematic	Video	Experts’ opinion
Pros	Lower dimensionality	Convenient to capture	Higher semantic level
	Actual 3D trajectories	Info. of the surroundings	
Cons	Needs tools	Higher dimensionality	Quantitative
	No info. of the surrounding	Estimated 3D trajectories	
		Occlusion, Clutter, etc.	
	Feature extraction technique		
	Hand-engineered	Automatic	
Pros	Interpretable	End to end solution	
	Easy to calculate	Case independent	
Cons	Hard to define	Requires a big dataset	
	Case dependent	Computational cost	

The most convenient hand-engineered features are those introduced by descriptive statistics Anh et al. (2020). In a skill rating system proposed by Brown et al. (2016), eight values of mean, standard deviation, minimum, maximum, range, root-mean-square (RMS), total sum-of-squares (TSS), and time integral of force and acceleration signals are calculated. Together with time features like task completion time, these values are used as inputs for a random forest classifier to rate the peg transfer score of 38 different participants. In Javaux et al. (2018), metrics like mean/maximum velocity and acceleration, tool path length, depth perception, maximum and integral of planar/vertical force, and task completion time are considered as a baseline for skill assessment Lefor et al. (2020). Another commonly used method in the literature is to use statistical tests such as Mann-Whitney test Moody et al. (2008), Kruskal–Wallis test Javaux et al. (2018), Pearson or Spearman correlation Zendejas et al. (2017), etc. These tests are utilized to classify the participants directly Moody et al. (2008) or automatically calculate some of the well-known skill assessment scores like GOALS and FLS Zendejas et al. (2017).

Since many surgical tasks are periodic by nature, the data frequency domain analysis proves to be effective Zia et al. (2015). For periodic functions like knot tying and suturing Zia et al. (2015) suggests that transforming the data into time series and performing a Discrete Fourier Transform (DFT) and Discrete Cosine Transform (DCT) on the data extracts features, will assist the skill level classification task. The results show that such an approach outperforms many machine-learning-based methods like Bag of Words (BoW) and Sequential Motion Texture (SMT). In another work by the same author, symbolic features, texture features, and frequency features are employed for the classification. A Sequential Forward Selection (SFS) algorithm is then utilized to reduce the number of elements in the feature vector and remove the irrelevant data Zia et al. (2016). Hojati et al. (2019) suggests that since Discrete Wavelet Transform (DWT) is superior to DFT and DCT in a sense that it offers simultaneous localization in time and frequency domain, DWT is a better choice for feature extraction in surgical skill assessment tasks.

As it is mentioned before, hand-engineered features are task-specific. For example, the frequency domain analysis discussed in the previous section is only viable when the task is periodic. Otherwise, the frequency domain features should be concatenated with other features. Moreover, perceiving the correct features that reflect participants’ skill levels in different surgical tasks requires an intensive knowledge of the field. As a result, developing a method in which the essential features are identified automatically is advantageous.

With the recent success of Convolutional Neural Networks (CNN) in classification problems like image classification, action recognition, and segmentation, it is safe to assume that CNN can be used in skill assessment problems. However, unlike image classification, improvement brought by end-to-end deep CNN remains limited compared to hand-engineered features for action recognition Wang et al. (2018). Similarly, using conventional CNN does not contribute too much to the result in surgical skill evaluation problems. For example, Fawaz et al. (2018) proposed a CNN-based approach for dry-lab skill evaluation tasks such as needle passing, suturing, and knot-tying. However, a hand-engineered-based method with a set of features introduced as holistic features (SMT, DFT, DCT, and Approximate Entropy (ApEn)) suggested by Zia and Essa (2018) reaches the same accuracy as the CNN-based method in the needle passing and suturing tasks and outperforms the CNN-based method in the knot-tying task.

Wang et al. (2018) suggests that conventional CNN falls short compared to traditional hand-crafted feature extraction techniques because it only considers the appearances (spatial features) and ignores the data’s temporal dynamics. In Wang and Fey (2018), a parallel deep learning architecture is proposed to recognize the surgical training activity and assess trainee expertise. A Gated recurrent unit (GRU) is used for temporal feature extraction, and a CNN network is used to extract the spatial features. The overall accuracy calculated for the needle passing, suturing, and knot tying tasks is 96% using video data. The problem of extracting spatiotemporal features is addressed with 3D ConvNets in Funke et al. (2019). In this method, inflated convolutional layers are responsible for processing the video snippets and unfolding the classifier’s input data.

To the best of our knowledge, all of the proposed methods in the literature have used single classifier techniques in their work. However, methods like classifier fusion have proved to be useful in the case of medical-related data. In Kazemian et al. (2005) an OWA-based fusion technique is used to combine multiple classifiers and improve the accuracy. For a more advanced classifier fusion technique, one can refer to the proposed method in Kazemian et al. (2010) where more advanced methods such as Dempster’s Rule of Combination (DCR) and Choquet integral are compared with more basic techniques. Activity recognition and movement classification is another efficient way to calculate metrics representing the surgical skill automatically Khan et al. (2020). Moreover, instrument detection in a video and drawing centroid based on the orientation and movement of the instruments can reflect the focus and ability to plan moves in a surgeon. Utilizing these centroids and calculating the radius, distance, and relative orientation can aid with the classification based on skill level Lavanchy et al. (2021).

**FIGURE 5:**
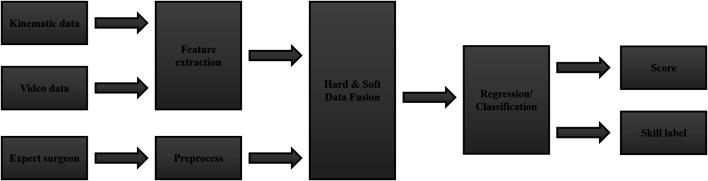
A general framework for surgical skill assessment.

In conclusion, the general framework illustrated in Figure 5 can summarize the reviewed techniques. The input data, either kinematic and video, is fed to a feature extraction block. A fusion block Naeini et al. (2014) can enrich the semantic of the data using expert surgeon feedback. Finally, a regression technique or a classifier can be employed to calculate a participant’s score based on his/her skill level or represent a label following his/her performance.

## 6 Machine Vision

The introduction of new hardware capable of running deep learning methods with acceptable performance led artificial intelligence to play a more significant role in any intelligent system Han (2017). It is undeniable that there is a huge potential in employing deep learning methods in a wide range of various applications Weng et al. (2019), Antoniades et al. (2016), Lotfi et al. (2018), Lotfi et al. (2020). In particular, utilizing a camera along with a deep learning algorithm, machines may precisely identify and classify objects by which either performing a proper reaction or monitoring a process may be realized automatically. For instance, considering a person in a coma, any tiny reaction is crucial to be detected, and since it is not possible to assign a person for each patient, a camera can solve the problem satisfactorily. Regarding the COVID-19 pandemic situation, artificial intelligence may be used to reduce both physical interactions and the risk of a probable infection especially when it comes to a medical training process. Considering eye surgery as an instance, not only should the novice surgeon closely track how the expert performs but also the expert should be notified of a probable mistake made by the novice surgeon during surgery. In this regard, utilizing computer vision approaches as an interface, the level of close interactions may be minimized effectively. To clarify, during the training process, the computer vision algorithm may act as both the novice surgeon looking over the expert’s hand and the expert monitoring and evaluating how the novice performs. This kind of application in a medical training process may easily extend to other cases. By this means, the demand for keeping in close contact is met properly.

Not needing a special preprocessing, deep convolutional neural networks (CNNs) are commonly used for classifying images into various distinct categories. For instance, in medical images, this may include probable lesions Farooq et al. (2017), Chitra and Seenivasagam (2013). Moreover, they can detect intended objects in the images which can be adopted not only to find and localize specific features but also to recognize them if needed. Since most of the medical training tasks require on-line and long-term monitoring, by utilizing a camera along with these powerful approaches, an expert may always keep an eye on the task assigned to a trainee. Besides, methods based on CNNs are capable of being implemented on graphics processor units (GPUs) to process the images with an applicable performance in terms of both speed and accuracy Chetlur et al. (2014), Bahrampour et al. (2015). This will reduce the probable latency and makes it possible for the trainer to be notified on time and correct the trainee remotely.

**FIGURE 6:**
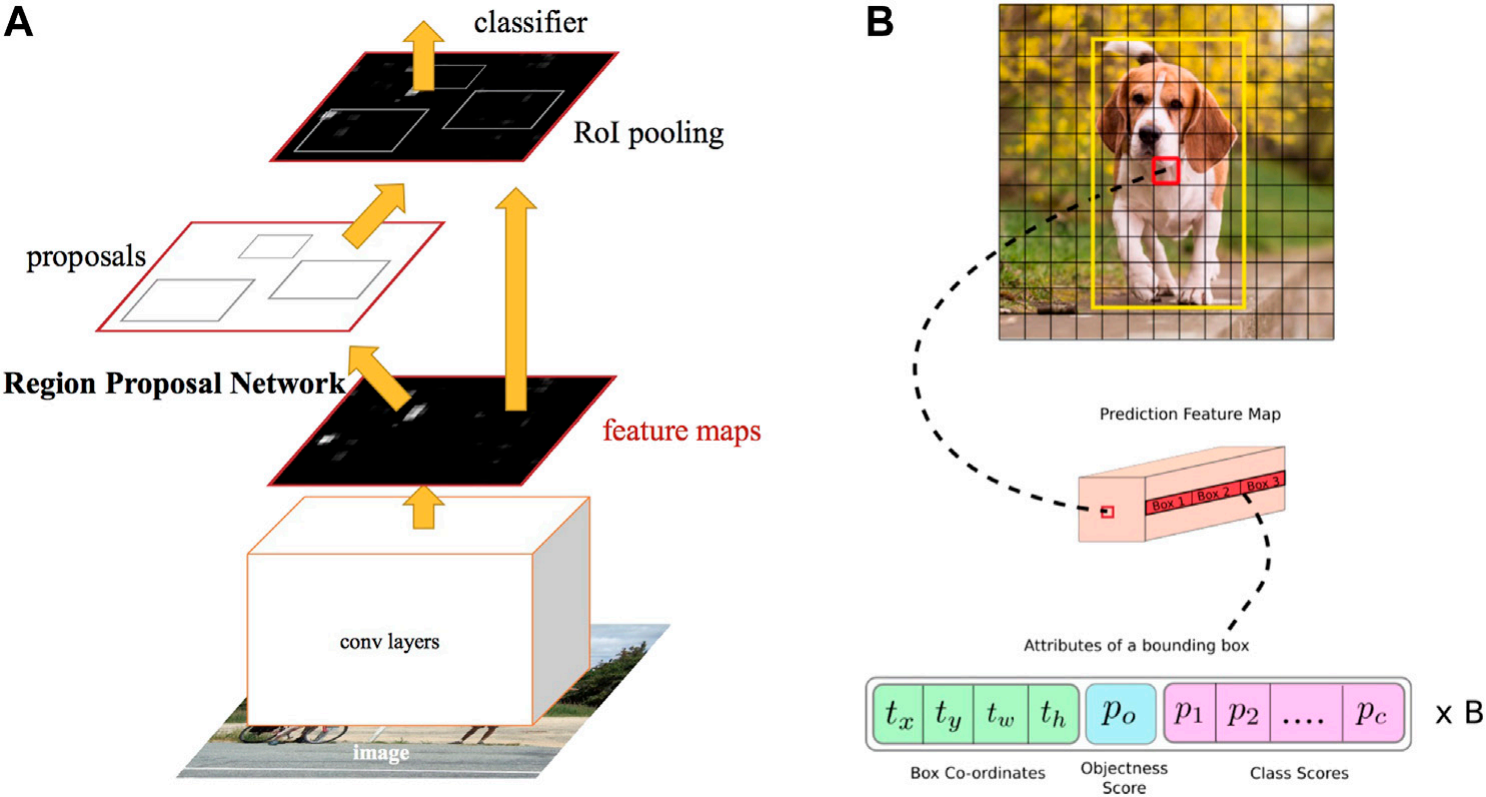
Example of two-stage and single-stage detectors Kathuria (2021). **(A)** Two-stage detector (RCNN). **(B)** Single-stage detector (YOLO).

There are numerous researches carried out in the field of image processing based on CNNs. These methods are mainly divided into two single-stage and two-stage detectors. The former is known to be fast while the latter results in higher accuracy. In Figure 6 the difference between a two-stage and a single-stage detector is illustrated. Considering single-stage detectors and starting with the LeCun et al. (1998) as one of the earliest networks, plenty of different approaches have been presented in the literature among which single-shot multi-box detector (SSD) Liu et al. (2016), RetinaNet Lin et al. (2017), and you only look once (YOLO) Redmon and Farhadi (2018) may be counted as nominated ones. Some of these approaches have been proposed with several structures including simpler and more complex structures to be employed depending on whether the speed is of high importance or accuracy. Mainly, training and the test are two phases when utilizing these methods. While it is crucial to define a proper optimization problem in the first phase, it is indispensable to implement the trained CNN optimally. Coming up with various solutions, methods like Krizhevsky et al. (2012), Simonyan and Zisserman (2015), Szegedy et al. (2015), and Szegedy et al. (2016) suggest utilizing specific CNN models to obtain better outcomes. On the other hand, to further improve the accuracy, in two-stage detectors like Girshick et al. (2014), it is suggested to first determine a region of interest (ROI) then identify probable objects in the related area. As a representative, Uijlings et al. (2013), which is known as selective search, is designed to propose 2*k* proposal regions, while a classifier may be employed for the later stage. Dealing with some challenging problems in these detectors, He et al. (2015), Girshick (2015), and Ren et al. (2015) are proposed to enhance the results in terms of both accuracy and speed.

To put all in a nutshell, when dealing with critical situations such as the current COVID-19 epidemic, it is highly recommended to employ artificial intelligence techniques in image processing namely deep CNNs for medical training tasks. By this means, neither is a close physical interaction between the expert and novice necessary, nor the quality of the training is reduced adversely due to the limitations. In fact, the computer vision approach acts as an interface making it possible both to learn from the expert and to evaluate the novice, remotely.

## 7 Conclusion and Future Prospects

The faculty members and the students of the medical universities are classified in the high-risk category due to the potential exposure to coronavirus through direct contact and aerosol-generating procedures. As a result, many medical schools have suspended their clinical programs or implemented social distancing in their laboratory practices. Furthermore, the current fight against the COVID-19 virus have used nearly all capacity of health-care systems, and some less urgent and less emergent medical services including the education issues are limited or even paused. Therefore, unless some assistive training tools are utilized to support the educational procedures, the training efficiency of medical universities will be reduced and it have future consequences for the world health-care system.

Practicing medical tasks with current lock-down policies can be solved utilizing state of the art techniques in haptics, virtual reality, machine vision, and machine learning. Notably, utilization of the above technologies in medical education has been researched actively within the past years in order to increase the safety and efficiency of the surgical training procedures. Nowadays, another motivation is created for those assistive technologies owing to the COVID-19 pandemic. In this paper, the existing assistive technologies for medical training are reviewed in the COVID-19 context and a summary of them is presented in Table 2.

**TABLE 2:** The main tools and approaches that help to reduce physical contact in medical training.

Training tool or technology	Approach	Some investigations
Virtual Reality	VR Based surgical training system	Bartlett et al. (2020), Cecil et al. (2018), Lohre et al. (2020), Medellin-Castillo et al. (2020)
	AR Based surgical training system	Si et al. (2019)
Haptic Technology	Single haptic simulators	Tahmasebi et al. (2008), Wang et al. (2014), Spera et al. (2020)
	Dual haptic with fixed authority	Nudehi et al. (2005), Khademian and Hashtrudi-Zaad (2012), Shahbazi et al. (2014b), Motaharifar et al. (2016), Motaharifar et al. (2019b)
	Dual haptic with variable authority	Shahbazi et al. (2014a), Motaharifar et al. (2019a), Motaharifar and Taghirad (2020), Liu et al. (2020)
Data Driven Scoring	DDS using hand-engineered features	Brown et al. (2016), Javaux et al. (2018), Hojati et al. (2019), Lefor et al. (2020)
	DDS using automated feature extraction	Wang and Fey (2018), Funke et al. (2019), Khan et al. (2020), Lavanchy et al. (2021)
	Fusion techniques	Naeini et al. (2014), Kazemian et al. (2010)
Machine Vision	Single-Stage Detectors	Redmon and Farhadi (2018), Liu et al. (2016), Lotfi et al. (2018), Lotfi et al. (2020)
	Two-Stage Detectors	Girshick (2015), Ren et al. (2015)
	Classifiers	Simonyan and Zisserman (2015), Szegedy et al. (2016)

It is reviewed that a surgical simulator system including a VR/AR based graphical interface and a haptic interface is able to provide the circumstances of actual surgical operation for the medical students, without the necessity of attending the hospital environments. Furthermore, through augmenting the system with another haptic console and having a dual user haptic system, the opportunity of collaboration with and receiving guidance cues from an expert surgeon in a systematic manner is given to the trainees. In contrast to the traditional collaboration methodologies, the haptic-based collaboration does not require the physical contact between the involved people and the risk of infection is reduced. Assessment of the expertise level of the medical students is another element of each training program. The necessity of reducing physical contact during the COVID-19 pandemic have also affected the skill assessment methodologies as the traditional ways of skill assessment are based on direct observation by a trainer. In contrast, data-based analysis may be utilized as a systematic approach for skill assessment without any need of physical contact. In this paper, some of the ongoing methods in surgical skill evaluation have been reviewed.

Biomedical engineering technology has progressed by leaps and bounds during the past several decades and advancements in remote diagnostics and remote treatment have been considered as a leading edge in this field. For instance, the tele-surgery robotic-assisted da Vinci system have received a great deal of attention in the healthcare marketplace with more than 5 million surgeries in the last 2 decades DaVinci (2021). However, the rate of advancement in medical training, which usually follows traditional methods, has been considerably less than the other aspects of medical field, and modern training technologies have received fewer attention during the past several decades. While remote training and remote skill assessment technologies make relatively lower risk to the patient than remote diagnostics and remote treatment, the reason behind fewer attention to the former is the lack of sufficient motivations. It is hoped that the motivations created for those advanced medical training methods during the COVID-19 crisis are strong enough to continuously increase their utilization among the medical universities. Although wide utilization of those technologies needs a considerable extent of time, effort, and investment, immediate and emergent decisions and actions are required to widely utilize those potential techniques. Notably, all of the presented approaches and techniques are targeted to be utilized in the normal situations without any pandemic in order to provide safer and more efficient medical training. Therefore, even after the world recovers from this crisis, these techniques, tools, and approaches deserve more attention, recognition, investigation, and utilization. There needs to be a global awareness among the medical universities that haptic technology and virtual reality integrated with machine learning and machine vision provides an excellent systematic medical training apparatus that ensures the requirements of health-care systems to enhance the safety, efficiency, and robustness of medical training.

## Data Availability

The original contributions presented in the study are included in the article/Supplementary Material, further inquiries can be directed to the corresponding authors.

## Footnotes

1 Less invasive stabilization system

2 Orthoganthic surgery
